# CCNA2 Is a Prognostic Biomarker for ER+ Breast Cancer and Tamoxifen Resistance

**DOI:** 10.1371/journal.pone.0091771

**Published:** 2014-03-12

**Authors:** Tian Gao, Yong Han, Ling Yu, Sheng Ao, Ziyu Li, Jiafu Ji

**Affiliations:** 1 Key Laboratory of Carcinogenesis and Translational Research Ministry of Education, Department of Gastrointestinal Surgery, Peking University Cancer Hospital & Institute, Beijing, China; 2 Key Laboratory of Carcinogenesis and Translational Research Ministry of Education, Department of Biochemistry and Molecular Biology, Peking University Cancer Hospital & Institute, Beijing, China; 3 Department of Orthopedics, Renmin Hospital, Wuhan University, Wuhan, Hubei, China; INRS, Canada

## Abstract

Identification of effective prognostic biomarkers and targets are of crucial importance to the management of estrogen receptor positive (ER+) breast cancer. CCNA2 (also known as CyclinA2) belongs to the highly conserved cyclin family and is significantly overexpressed in various cancer types. In this study, we demonstrated that CCNA2 had significant predictive power in distant metastasis free survival, disease free survival, recurrence free survival and overall survival of ER+ breast cancer patients. We also found that CCNA2 was closely associated with tamoxifen resistance. In addition, gene set enrichment analysis (GSEA) revealed that its expression was positively associated with genes overexpressed in endocrine therapy resistant samples. Finally, though CCNA2-Drug interaction network, we demonstrated the interactions between CCNA2 and several available cancer drugs. Overall, we suggest that CCNA2 is a biomarker for the prognosis of ER+ breast cancer and monitoring of tamoxifen efficacy. It's also a promising target for developing new strategies to prevent or even reverse tamoxifen resistance. Moreover, CCNA2 expression may help monitoring tamoxifen efficacy and directing personalized therapies. Nevertheless, in vivo and in vitro experiments and multi-center randomized controlled clinical trials are still needed before its application in clinical settings.

## Introduction

Breast cancer is the most common malignancy among women in the United States, among which 70% of them are ER+. The selective ER modulator tamoxifen has shown great success in the treatment of ER+ breast cancer[1]. However, over 40% ER+ patients with advanced disease fail to respond to tamoxifen effectively, even for those who responded at the beginning would develop acquired resistance eventually[2]. Approximately 25% of all women diagnosed with breast cancer die from their disease despite having been treated according to state-of-the-art clinical guidelines[3]–[5]. In the meantime, adjuvant systemic therapy saves a significant number of lives[6]–[8], however, many patients are subjected to unnecessary adjuvant therapies with the potential of causing more harm than good[9]. The present lack of criteria to help individualize breast cancer treatment indicates the need for a novel way to predict prognosis and therapy response. Since about one-half of the patients with estrogen receptor- positive cancer fail on tamoxifen[10], [11], identification of effective and reliable biomarkers that could be used to monitor tamoxifen efficacy and new targets to reverse tamoxifen resistance is of crucial importance.

CCNA2 (also known as CyclinA2) belongs to the highly conserved cyclin family and is expressed in almost all tissues in human body[12]. It plays critical roles in the control of cell cycle at the G1/S and the G2/M transitions and is essential in embryonic cells and in the hematopoietic lineage[13]. Data from Human Protein Atlas show that CCNA2 is overexpressed in dozens of cancer types, which indicates its potential roles in cancer transformation and progression[14]. It is also reported that CCNA2 may be involved in the processes of epithelial-mesenchymal transitions (EMT) and metastasis[15].

Nevertheless, the prognostic power of CCNA2 in ER+ breast cancer and its relation with tamoxifen resistance have never been reported before.[16] In this study, we explored the possibility of CCNA2 as a biomarker for the prognosis of ER+ breast cancer patients and prediction of tamoxifen efficacy. Besides, an interaction network was constructed to show how CCNA2 and available anti-cancer drugs could interaction with each other.

## Materials and Methods

### Ethics statement

We have the right to use datasets from Gene Expression Omnibus (GEO) by complying with all requirements according to each dataset. The Research Ethics Committee of Peking University Cancer Hospital & Institute waived the requirement for ethical approval of this analysis because the registry is a de-identified database.

### Patients and cell lines

Several publicly available datasets were downloaded and analyzed to explore the prognostic value of CCNA2 in ER+ breast cancer patients and its potential role in tamoxifen resistance.

GSE47561 contains expression data from 1570 breast cancer samples, of them, 514 ER+ patients with distant metastasis free survival (DMFS) data (114 of them are known to be treated with tamoxifen), 513 ER+ patients with recurrence free survival (RFS) data, 125 ER+ patients with disease free survival (DFS) and overall survival (OS) data, 167 ER- patients with DMFS data. Van cohort contains expression data from 226 ER+ breast cancer samples that with DMFS, DFS and OS data. Gyorffy dataset (256 samples) and GSE3494 (83 samples) are expression data from ER+ breast cancer samples that with DMFS data and are known to be treated with tamoxifen. ISDB3008 dataset contains expression data from 2795 breast cancer samples and is obtained through InsilicoMerging package[17] in R 3.0.1. Raw data (*.CEL) in this dataset was normalized using FRMA method and all the probes were mapped to gene symbols. GSE33366 contains expression data from MCF-7 tumor xenografts treated with tamoxifen or control for 28 days, each group with 2 biological replicates. GSE26459 contains expression data from subcloned MCF-7 cell lines that were either highly sensitive or naturally resistant to tamoxifen, each group with 3 biological replicates.

Among all the datasets used in this study, GSE47561, GSE3494, GSE33366 and GSE26459 are obtained from Gene Expression Omnibus (GEO) dataset; van dataset and Gyorffy dataset were obtained from supplementary data of previous publications. For all the study subjects in this research, ER positivity was defined as greater than 10 fmol/mg tumor tissue and greater than 1% nuclear staining or immunohistochemistry (IHC) score of at least 3 for the biochemical and immunohistochemical assays, respectively.

### Genomic analysis

The mRNA expression profiling of all the samples in this study were performed on the Human U133A Gene Chip or Human genome U133 plus 2.0 platforms (Affymetrix, Santa Clara, CA). GSE47561[18], Van dataset[18], Gyorffy dataset[19], and GSE3494[20] were used for survival analysis. Patients were classified into three groups according to the tertiles of CCNA2 expression values in Kaplan-Meier plots for DMFS, DFS and RFS. This classification method could show the dynamic changes of the probability that patients would remain free of distant metastasis, disease and recurrence according to different CCNA2 expression levels (high, middle and low). In the Kaplan-Meier plot for OS and DMFS (tamoxifen treated subsets from GSE47561, GSE3494 and Gyorffy dataset), patients were classified into two groups according to the upper tertile of CCNA2 expression values. This classification method could show the survival probability differences between high CCNA2 group and low CCNA2 group with more significance.

GSE33366 and GSE26459[21] were used to show the association between CCNA2 expression and tamoxifen efficacy. Gene Set Enrichment Analysis (GSEA)[22] was performed using expression data of GSE47561 and ISDB3008 on gene sets related with tamoxifen resistance including ‘ENDOCRINE THERAPY RESISTANCE’ and ‘TAMOXIFEN RESISTANCE DN’ etc. The Comparative Toxicogenomics Database (CTD)[23] was explored to construct CCNA2-drug interaction network. More specifically, CCNA2 was searched in the CTD database for drugs or chemicals that could decrease/increase the mRNA or protein expression of CCNA2. Then drugs or chemicals were selected based on their applications in breast cancer management. Finally, CCNA2-drug interaction network was constructed through Cytoscape version 3.0.2.

### Statistical analysis

Clinical and gene expression profiling data were analyzed using standard statistical tests including the logrank test and unpaired t-test. In GSEA, we assess the significance of an observed enrichment score (ES) by comparing it with the set of scores ES_NULL_ computed with randomly assigned phenotypes. The nominal p value estimates the statistical significance of the enrichment score for a single gene set. A P value of zero (0.0) indicates an actual P value of less than 1/number-of-permutations. In the present study, we performed 1000 permutations. Significance was defined as a P value of less than 0.05. Analyses were performed using R 3.0.1 (R Foundation for Statistical Computing [http://www.r-project.org/]), GraphPad Prism 5.01 (GraphPad Software, Inc. [www.graphpad.com]) and SPSS version 21 (SPSS Inc, Chicago, Illinois).

## Results

### CCNA2 is a prognosis biomarker in ER+ breast cancer

**Figure 1:**
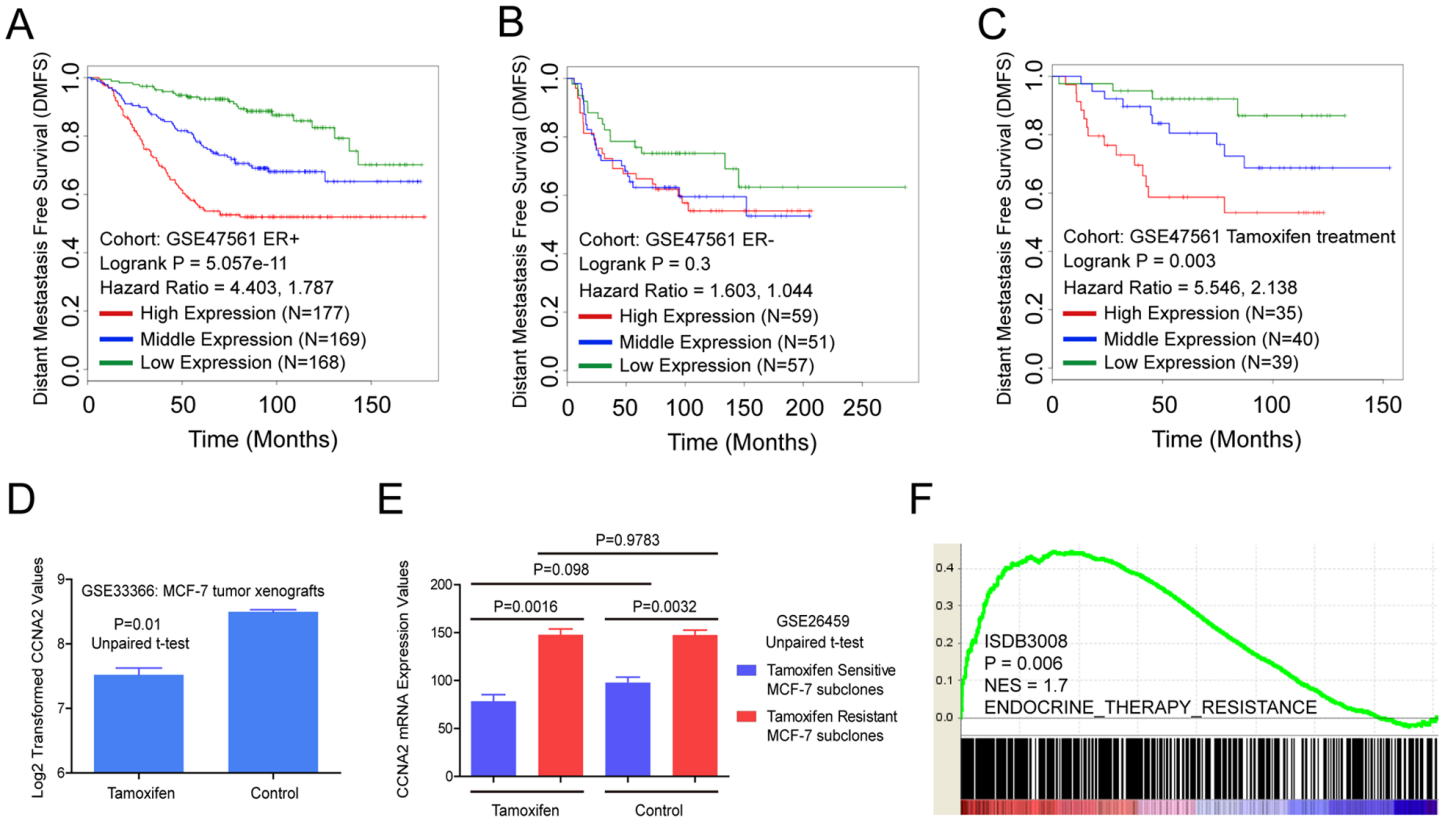
CCNA2 expression is associated with poor survival and tamoxifen resistance in ER+ breast cancer patients. Kaplan-Meier plot for DMFS of ER+ (*A*), ER- (*B*) or tamoxifen treated (*C*) breast cancer patients grouped by the tertile of CCNA2 expression levels, significance was assessed by logrank test. (*D*) Log2 transformed mRNA expression values of CCNA2 in MCF-7 tumor xenografts treated with either tamoxifen or control. P values were calculated by unpaired two-tailed t test. Error bars represent mean ± SEM. (*E*) mRNA expression values of CCNA2 in tamoxifen resistant/sensitive MCF-7 subclones treated with tamoxifen or control. P values were calculated by unpaired two-tailed t test. Error bars represent mean ± SEM. (*F*) Gene set enrichment analysis of CCNA2 mRNA expression in relation to tamoxifen resistance gene set using breast cancer expression profiles (N = 2795).

By analyzing gene expression profiles and corresponding clinical information of breast cancer patients from publicly available datasets, we found that high expression of CCNA2 confers poor distant metastasis free survival (DMFS), disease free survival (DFS), recurrence free survival (RFS) and overall survival (OS) in ER+ breast cancer patients (Fig. 1A, Fig. S1). However, the expression levels of CCNA2 are not correlated with DMFS in ER- breast cancer patients (Fig. 1B).

Specifically, CCNA2 overexpression is associated with poor distant metastasis survival in ER+ breast cancer cohorts GSE47561 (Fig. 1A, P = 5.057e−11) and Van dataset (Fig. S1A, and 8.093e−05). Besides, overexpression of CCNA2 also confers poor disease free survival (Fig. S1B, P = 2.433e−06 and Fig. S1C, P = 3.583e−05), relapse free survival (Fig. S1D, P = 2.601e−08) and overall survival (Fig. S1E, P = 2.03e−04 and Fig. S1F, P = 0.038) in ER+ breast cancer cohorts as noted in each figures. However, the association between CCNA2 expression levels and DMFS in ER- breast cancer patients is not significant (Fig. 1B, P = 0.3).

All these data indicates that CCNA2 confers poor prognosis in ER+ breast cancer.

### CCNA2 is a biomarker for tamoxifen resistance

Since high expression of CCNA2 confers poor DMFS, DFS, RFS and OS in ER+ breast cancer patients and tamoxifen is one of the most widely used drugs for the management of those ER+ patients, CCNA2 may play a potential role in tamoxifen resistance. To validate this hypothesis, several datasets were downloaded from publicly available datasets and analyzed. Results showed that high expression of CCNA2 was significantly associated with poor tamoxifen efficacy in ER+ breast cancer within GSE47561, Gyorffy et al. and GSE3494 (Fig. 1C, Fig. S2A and S2B, P = 0.003, 0.028 and 0.01, respectively).

In vivo data showed that CCNA2 expression was down-regulated in MCF-7 tumor xenografts treatment with tamoxifen compared with control (Fig. 1D, P = 0.01), which indicates that tamoxifen could decrease CCNA2 expression in ER+ breast cancer cells. In vitro experiment results showed that CCNA2 was overexpressed in tamoxifen resistant MCF-7 subclones compared with sensitive controls regardless of tamoxifen treatment or not (Figure 1E, P = 0.0016 and 0.0032, respectively), which indicates CCNA2's potential role in tamoxifen resistance. It can be revealed that tamoxifen could down-regulate CCNA2 expression in vivo (Figure 1D, P = 0.01). Similar trends are emerging in vitro (Figure 1E, P = 0.098), however, it has no statistic significant. Moreover, it has no influence on CCNA2 expression in tamoxifen resistant subclones (P = 0.9783).

Besides, Gene Set Enrichment Analysis performed on expression data from ISDB3008 (2795 samples) indicated that CCNA2 expression was positively correlated with genes overexpressed in endocrine therapy resistant samples (Fig. 1F, P = 0.006). But data from GSE47561 (1570 samples) displayed a slight but not significant increase (Fig. S2C, P = 0.12). Further GSEA analysis showed that the expression of CCNA2 was negatively correlated with genes that down-regulated in tamoxifen resistant samples (Fig. S2D, P = 0.008).

All these data suggests that CCNA2 expression is correlated tamoxifen resistance.

### CCNA2-Drug interaction network indicates drugs that could decrease CCNA2 expression

**Figure 2:**
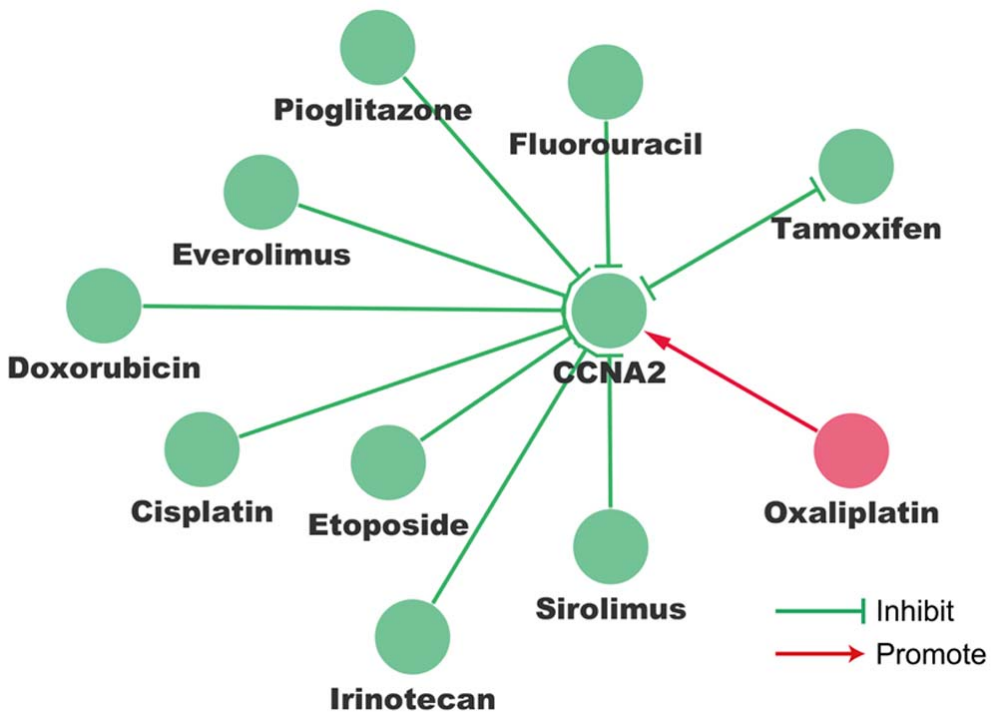
Interaction network of CCNA2 and chemotherapeutic drugs. The gene-drug interaction network shows us how available chemotherapeutic drugs could decrease the expression of CCNA2. For example, Doxorubicin could decrease the expression of CCNA2, while Oxaliplatin could increase the expression of CCNA2.

Next, we sought to explore how CCNA2 and available cancer drugs could influence each other. CCNA2-Drug interaction network was constructed in Cytoscape based on data from The Comparative Toxicogenomics Database (CTD) (Fig. 2). Please see Methods section for procedure details. This network indicates that several drugs could influence the mRNA or protein expression of CCNA2. For instance, Doxorubicin and Everolimus could decrease CCNA2 expression while Oxaliplatin could increase CCNA2 expression. Each arrow in this network is supported by previous reports.

## Discussion

The therapy of ER+ breast cancer, which represents more than 70% of breast tumors, is based on anti-hormonal compounds[24]. The anti-estrogen tamoxifen is a commonly used treatment for patients with ER+ breast cancer. For adjuvant therapy of ER+ breast cancer, tamoxifen improves overall survival and reduces risk for development of breast cancer [25]. Unfortunately, a subset of patients who received adjuvant tamoxifen would eventually experience relapse and die as a result of the disease, 30% of ER+ tumors were not prevented by tamoxifen in National Surgical Adjuvant Breast and Bowel Project(NSABP) prevention trial(P1) [26].

Numerous studies have been performed, which combined endocrine therapy with agents that could modulate these mechanisms, so as to prevent the occurrence of tamoxifen resistance[27]. Due to the pressing clinical need, several other investigators have developed gene predictors that c predict outcome in ER+ breast cancer treated with adjuvant tamoxifen therapy. For instance, Cyclin D1, Acid ceramidase 1and p53 accumulation had been reported that could predict outcome in ER+ breast cancer treated with adjuvant anti-estrogen therapy [28]–[34]. Likewise, Retinoic acid receptor alpha, CD44 and deltaEF1 had been reported to be involved in the development of tamoxifen resistance in breast cancer. [35]–[37]. It have been reported that breast stem cells and Wnt signaling activation may be the mechanism of resistance to tamoxifen[38], [39].

CCNA2, a key regulator of cell cycle, is overexpressed in many human cancers, including breast cancer. However, the association between CCNA2 overexpression and tamoxifen resistance in ER+ breast cancer remains unclear. Our results show the significant prognostic power of CCNA2 in ER+ breast cancer progression and tamoxifen resistance. Importantly, high level of CCNA2 is correlated with tamoxifen treatment failure and poor DMFS. We might be able to stratify ER+ breast cancer patients by testing the mRNA expression level of CCNA2and decide when and how to use tamoxifen treatment in combination with appropriate therapeutic drug in the future. Moreover, we demonstrated that CCNA2 is down-regulated in MCF-7 tumor xenografts treated with tamoxifen compared with control in vivo. However, tamoxifen has no significant influence on CCNA2 expression in tamoxifen resistant subclones (P = 0.9783), which indicates CCNA2's potential role in tamoxifen resistance. Nevertheless, whether CCNA2 is a driver gene in tamoxifen resistance still requires experimental validation.

Provided the high expression of CCNA2 is involved in the development of tamoxifen resistance, how to manage cancer patients with CCNA2 overexpression remains a great challenge. Here we show several drugs that could influence the expression of CCNA2 (Fig. 2). For instance, Doxorubicin could decrease the expression of CCNA2, while Oxaliplatin could increase the expression of CCNA2. However, whether ER+ breast cancer patient with CCNA2 overexpression could benefit from the repression of CCNA2, or in other words, whether CCNA2 is a promising target for preventing or could reverse tamoxifen resistance still needs more experimental support.

Although much information about ER and cancer has been provided in the past three decades since the arrival of tamoxifen in the clinic, a lot more needs to be elucidated for favorable therapeutic outcomes. More concrete research outcomes will warrant the translational research that may lead to more efficient and safer treatment for breast cancer patients as well as women at high risk of advanced breast cancer.

Taken together, this study indicates that CCNA2 expression may help monitoring tamoxifen efficacy. In addition, it suggests the relevance of CCNA2 in the development of tamoxifen resistance. Furthermore, it could provide guidance personalized therapies. Nevertheless, multi-center randomized controlled clinical trials and in vivo/in vitro experiments are still needed before its application in clinical settings.

## Supporting Information

Figure S1 **Kaplan-Meier plot for DMFS (** ***A*** **), DFS (** ***B*** **and** ***C*** **), RFS(** ***D*** **) of ER+ breast cancer patients classified according to the tertile of CCNA2 expression levels.** (*E* and *F*) Kaplan-Meier plot for OS of breast cancer pateints classified according to the tertile of CCNA2 expression level. Significance was assessed by logrank test.
(TIF)

Figure S2 **(** ***A*** **and** ***B*** **) Kaplan-Meier plot for DMFS of tamoxifen treated breast cancer patients classified according to the tertile analysis of CCNA2 expression level, significance was assessed by logrank test.** Gene set enrichment analysis of CCNA2 mRNA expression in relation to gene set up-regulated in endocrine resistant patients (C) and gene set down-regulated in tamoxifen resistant MCF-7 cells (D) using breast cancer expression profiles GSE47561.
(TIF)
