# Supplementary figures and images for: CCNA2 Is a Prognostic Biomarker for ER+ Breast Cancer and Tamoxifen Resistance
Source: PLoS One. 2014 Mar 12;9(3):e91771. doi: 10.1371/journal.pone.0091771 (PMC3951414; doi:10.1371/journal.pone.0091771)

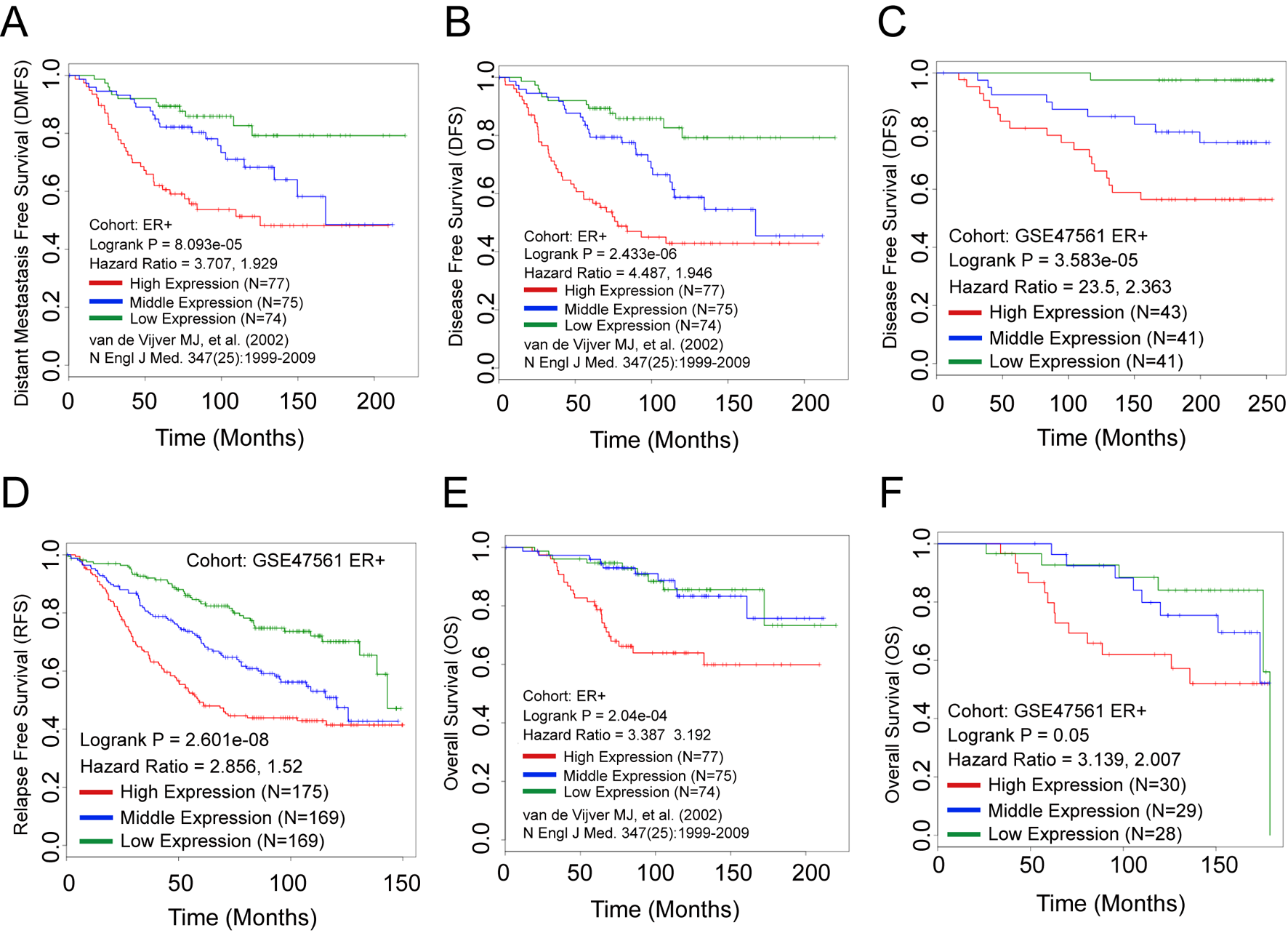

Supplement: Figure S1 — Kaplan-Meier plot for DMFS ( A ), DFS ( B and C ), RFS( D ) of ER+ breast cancer patients classified according to the tertile of CCNA2 expression levels. (E and F) Kaplan-Meier plot for OS of breast cancer pateints classified according to the tertile of CCNA2 expression level. Significance was assessed by logrank test. (TIF) [file pone.0091771.s001.tif]

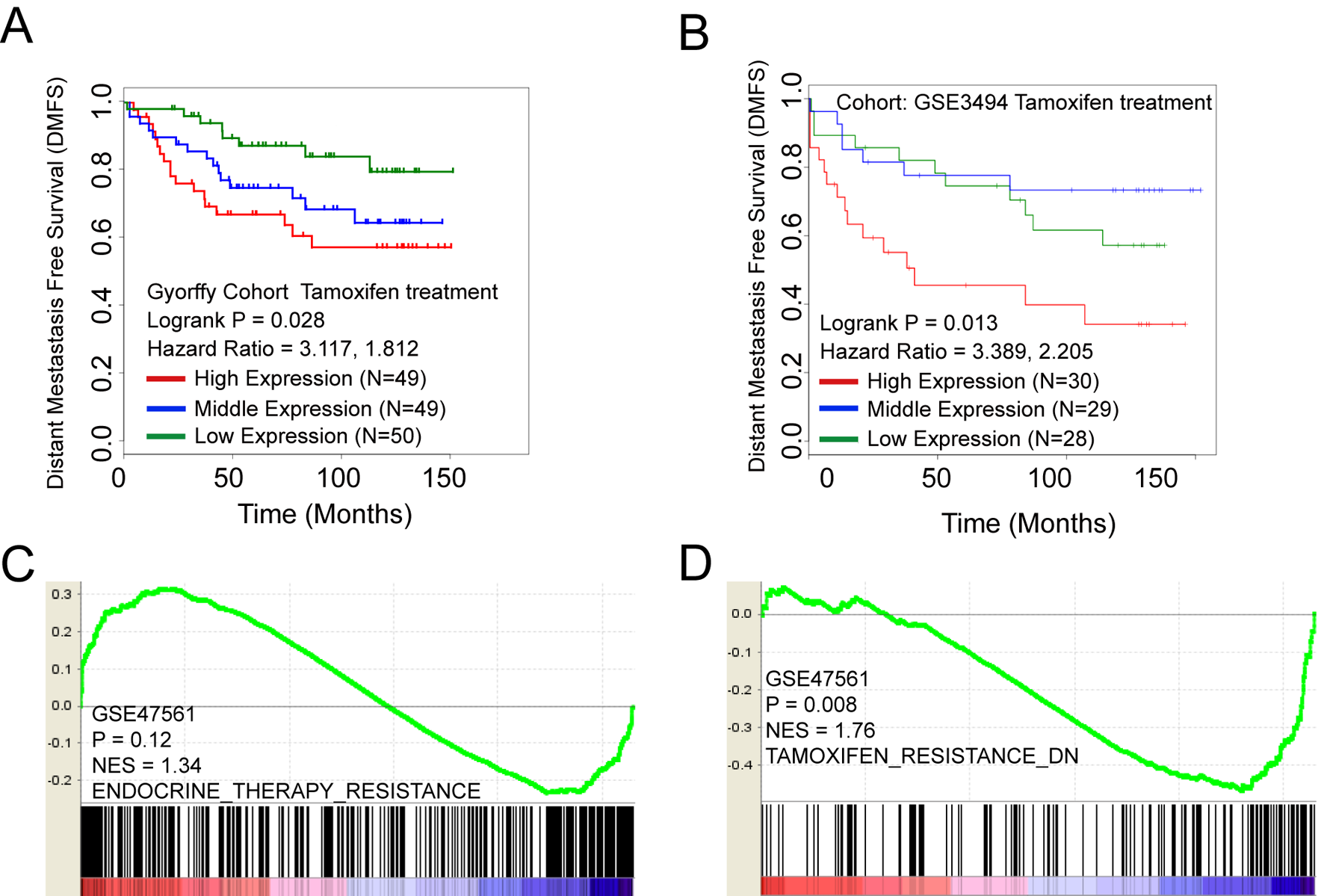

Supplement: Figure S2 — ( A and B ) Kaplan-Meier plot for DMFS of tamoxifen treated breast cancer patients classified according to the tertile analysis of CCNA2 expression level, significance was assessed by logrank test. Gene set enrichment analysis of CCNA2 mRNA expression in relation to gene set up-regulated in endocrine resistant patients (C) and gene set down-regulated in tamoxifen resistant MCF-7 cells (D) using breast cancer expression profiles GSE47561. (TIF) [file pone.0091771.s002.tif]
